# The role of life experience in affecting persistence: A comparative study between free-ranging dogs, pet dogs and captive pack dogs

**DOI:** 10.1371/journal.pone.0214806

**Published:** 2019-04-17

**Authors:** Martina Lazzaroni, Friederike Range, Lara Bernasconi, Larissa Darc, Maria Holtsch, Roberta Massimei, Akshay Rao, Sarah Marshall-Pescini

**Affiliations:** 1 Wolf Science Center, Domestication Lab, Konrad Lorenz Institute of Ethology, University of Veterinary Medicine, Vienna, Austria; 2 Comparative Cognition, Messerli Research Institute, University of Veterinary Medicine, Vienna, Austria; Memorial University of Newfoundland, CANADA

## Abstract

Persistence in object manipulation has been consistently associated with problem-solving success and it is known to be affected, at the individual level, by life experience. Differences in life experiences are particularly poorly studied in the problem-solving context and mainly refer to the comparison between wild and captive animals. Dogs represent interesting study subjects, since dog populations differ widely in their life experiences. In this comparative study we investigated subjects' persistence when presenting a novel object containing food that could not be accessed (impossible task) to three dog populations with very diverse life experiences: free-ranging village dogs (in Morocco), pet dogs (in Vienna) and captive pack living dogs (Wolf Science Center-WSC). We found that pet dogs and captive dogs (WSC) were more manipulative and persistent than free-ranging dogs. The low persistence of free ranging-dogs is unlikely the effect of a lack of exposure to objects, since they are confronted with many human’ artefacts in their environment daily. Instead, we suggest that the higher persistence of captive dogs and pet dogs in comparison to free-ranging dogs might be due to their increased experience of human-mediated object interaction. This provides subjects with a socially guided experience in manipulating and interacting with objects increasing their motivation to engage in such tasks.

## Introduction

Problem solving performance is a cognitive trait which is an accepted measure of general cognitive ability [1] and has been observed to variably influence subjects' fitness [2–5]. Studies investigating the factors that influence individuals' problem-solving performance focus on both intra-specific (for review see [6]) and inter-specific [7–11] comparison. In both cases, subjects' performance has been observed to be determined by a combination of numerous factors, i.e. innovation [8, 12–14], neophobia [7, 12–14], behavioural flexibility [12, 15], and persistence or task-directed motivation [8, 12–14]. Moreover, in intra-specific comparisons, problem solving performance seems to be additionally influenced by factors such as personality [14, 16], social rank [14], sex [12, 17], age [8, 12, 14, 18, 19], and individual life experience [20–22].

Differences in life experiences are particularly poorly studied in the problem-solving context and mainly refer to the comparison between wild and captive animals. Here, captive animals have been found to outperform their wild counterparts when presented with problem-solving tasks [20–22], which could be due to them having a safer and more enriched environment (i.e. material conditions). This may allow them more opportunities to practice object-manipulation and be less neophobic towards human artefacts. Another possible explanation is that experience with humans leads to an increase in socially guided exploration opportunities that enhance captive animals’ problem-solving performance [23, 24].

Since the life experience of the individual may influence problem-solving performance, dogs represent interesting study subjects. In fact, dog populations differ widely in their life experiences. In terms of their socio-ecology, dogs are an anthrodependent species [25] that have adapted to live and reproduce around human settlements [26]. Classically, they are considered ‘pets’, despite this category representing only 20–30% of the world dog population [27]. Pet dogs are restricted dogs that fully depend on humans for food provisioning, reproduction, and movements [28, 29]. Their social lives are deeply interconnected with those of their human companions [30] with whom they establish complex social relationships [31]. The other 70–80% of the world dog population is represented by free-ranging dogs [27, 32, 33]. Despite being self-sustaining and self-regulating populations, without any [major] human constraints on their activities [34], free-ranging dogs mostly live around humans settlements in urban, suburban, and rural environments [35, 36]. Although ‘feral’ dogs that live removed from humans and base their survival on hunting small prey exist [27, 36–38], the majority of free-ranging dogs are largely scavengers [39–45] that rely on resources provided by human activity [45]. Free-ranging dogs living in urban areas have a close association with humans and tend to be solitary or form smaller groups compared to those living in rural areas that are more independent from humans and tend to form bigger groups [35, 36] with a well-defined social structure [46, 47].

Despite the evidence that life experience may influence individuals' problem-solving performance, only a few studies have investigated problem-solving abilities across dog populations with different experiences. Many of these studies focused on pet dogs, comparing trained and untrained dogs [48–52]. The main finding of these studies is that training (i.e. experience in interacting with objects with human guidance) improves subject's persistence and problem-solving success (but see [52]).

In a study on problem-solving performance in dogs and wolves, Udell et al. [53] additionally compared two dog populations with different life experiences: shelter dogs and pet dogs. The authors did not find differences between the two dog groups, which both showed low persistence and problem-solving success. Interestingly, they found that for both groups the subject's persistence to interact with the puzzle box was higher when encouraged by the human than when tested alone. They suggested that the low persistence showed by the subjects when tested alone was due to the experience of being inhibited by the owners in interacting with the objects. In fact, it is commonly assumed that the poor performance of dogs in problem-solving tasks is a consequence of the fact that dogs' day-to-day behaviours are often regulated by their owners [53]. Thus, dogs may be inhibited to interact with the objects or just avoid to solving problems because they are used to receiving help from the human partner [54]. However, shelter dogs and pet dogs behaved similarly in this task, which was explained by the authors as social inhibition being rather generalized. In contrast to this hypothesis are the findings of another study also comparing problem-solving performance between shelter dogs and pet dogs [55]. In this study, Barrera et al. [55] found that pet dogs where more persistent in looking for a reward than shelter dogs. Moreover, they did not observe differences in persistence between dogs tested alone or with the experimenter. This would exclude that the lower persistence of shelter dogs was due their higher needs to socially interact with a human instead of focusing on the task.

The contrasting results observed in these studies may be due to the fact that the past experience of shelter dogs with humans could be extremely variable. This could be due to different shelter-management styles or a difference in dogs’ experiences, since some may have lived for years as pet dogs and others as free-ranging dogs. Thus, to properly test the effect of the experience with humans it would be necessary to compare dogs that strictly depend on humans (i.e. pet dogs and captive dogs) with unrestricted free-ranging dogs.

To our knowledge, only one study has investigated differences in problem-solving between pet dogs and free-ranging dogs [56]. Interestingly, the study found that free-ranging dogs were less persistent (i.e. they interacted with the apparatus for a shorter duration) than pet dogs. This is in line with what was observed in other species where captive animals outperform their wild counterparts [20–22]. However, this result was restricted to the condition when the pet dogs were tested indoors; when they were tested outdoors no difference emerged between pet and free-ranging dogs due to a decrease in the pet dogs’ persistence, probably because of lower motivation in this context. Furthermore, the experimenter’s/owner’s presence during the test may have influenced the behaviour of the subjects differently, with free-ranging dogs potentially being more nervous in the presence of the human than pets.

In the current study we investigated differences in persistence between dog groups differing in their experience with humans. We tested free-ranging dogs and pet dogs in an impossible task (i.e. an object containing food that could not be accessed), in their home environment. We used the same paradigm used by a previous study conducted on captive dogs and wolves at the Wolf Science Center (WSC) [57]. Additionally, we compared our data from pet and free-ranging dogs with the previous data on enclosure-kept dogs that differ in their experience from pet dogs. We investigated whether persistence is affected by the subject’s experience and hypothesised that the experience interacting with humans and their artefacts would increase subjects’ persistence.

All subjects were tested with a big rigid plastic ball that they had never encountered before (however, all dogs might have been exposed to ball-like objects before). Additionally, as neophobia (defined as 'the avoidance of an object solely because it has never been experienced and is dissimilar from what has been experienced in the individual's past’ [58]) has often been shown to negatively correlate with persistence ([12, 14, 59] but see [60]), we also tested both free-ranging dogs and pet dogs with a plastic bottle–a familiar object that all subjects have considerable experience with. This allowed us to assess the potentially different effect of neophobia on persistence in the two dog populations. All tests were performed in the absence of conspecifics and humans.

We predicted that if persistence is influenced by different life experiences with humans as observed in other species [20–22], WSC dogs and pet dogs should be more persistent with the ball than free-ranging dogs. Additionally, pet dogs (that live in constant contact with humans) should be more persistent with the ball than WSC (and free-ranging) dogs.

Finally, since the population of free-ranging dogs that we tested has been observed to live around a wide variety of human artefacts, we expected neither free-ranging dogs nor pets to act differently when tested with the ball compared to the plastic bottle.

## Materials and methods

### Ethical statement

Ethical approval for this study was obtained from the ‘Ethik und Tierschutzkommission’ of the University of Veterinary Medicine (Protocol number ETK-15/05/2016). Informed consent was obtained by all owners of the pet dogs. The authorization to test the free-ranging dogs was provided by the municipality of Taghazout (Morocco).

### Subjects

#### Free-ranging dogs (FRd)

**Fig 1:**
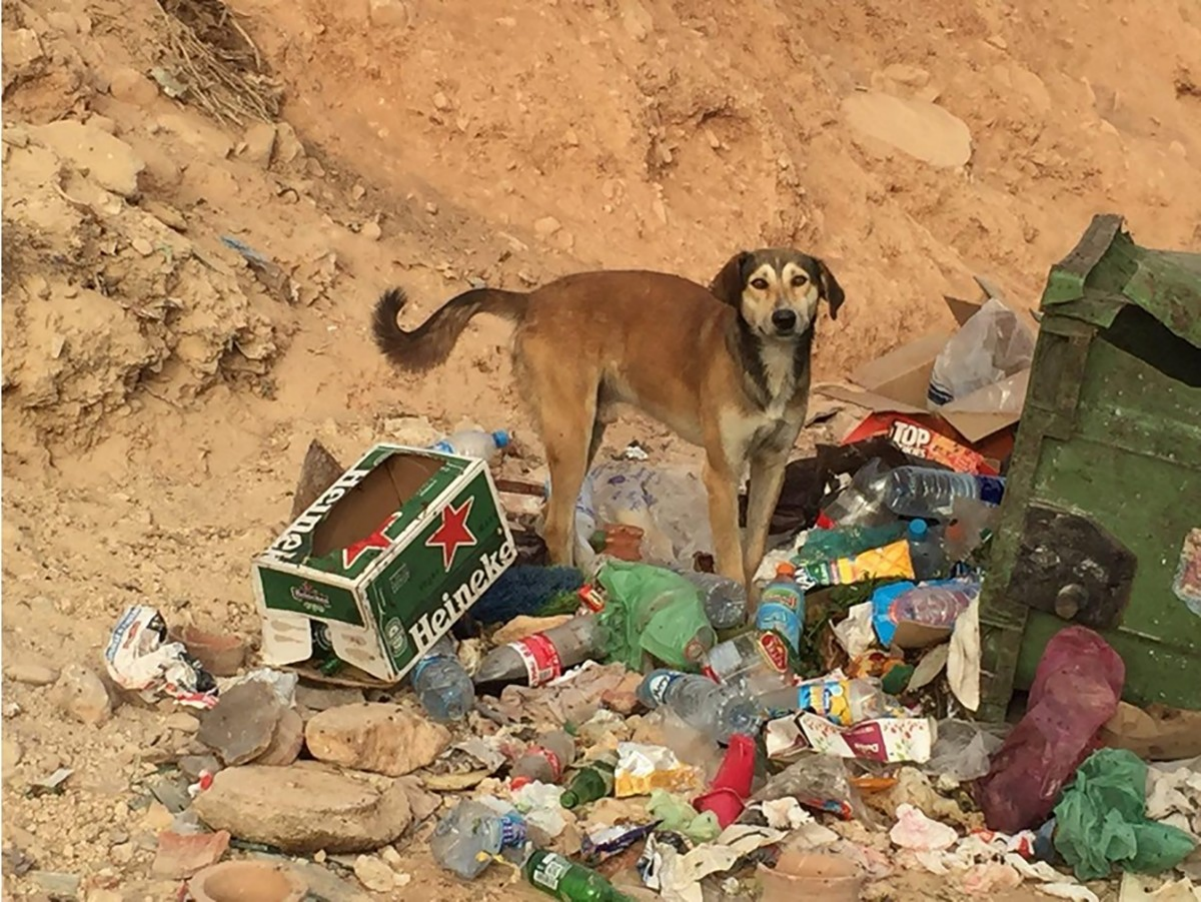
Free ranging dog looking for food in the garbage. In the study area the experience of finding food in the garbage, surrounded by human's artefacts (such as plastic bottles, boxes, bags, etc..) is common since early age.

Free-ranging dogs were tested in their natural environment in the municipality of Taghazout, Agadir, Morocco. The experimenters (ML, LD and RM) travelled by car to look for solitary dogs (solitary dogs were chosen to avoid interference by conspecifics). Only adult dogs (appearing to be over 1 year of age) were tested. A total of 35 free-ranging dogs were tested, 32 (20 F, 12 M) were included in the analyses (10 dogs were tested with both the ball and the bottle, 13 with only the ball and 12 with only the bottle). Three dogs were excluded because did not approach the apparatus. The tested free-ranging dogs were village-dogs living around human settlements. Their main source of food is derived from human waste that is both concentrated in specific spots (i.e. bins) and scattered around, especially during the tourist seasons (Fig 1).

#### Pet dogs (Pd)

Mixed-breed pet dogs were tested in private gardens in Austria. A total of 25 pet dogs were tested; 24 were included in the analyses (15F, 9M mean age: 7 years; range: 1 to 15). One dog was excluded because did not approach the apparatus. Four tests with the bottle were stopped before the end due to the owner’s request (but were kept for analyses, see the result section). Twenty-two dogs were tested with both the ball and the bottle, 2 with only the ball and 1 with only the bottle.

#### Enclosure kept, pack-living dogs (WSCd)

16 mixed-breed dogs (8F, 8M: mean age in years: 4, range: 2 to 6) housed at the Wolf Science Center (www.wolfscience.at) were tested. Dogs live in conspecific packs and are hand raised. The animals are trained and participate in behavioural tests (for further information on this population see [61]). All animals were presented with the ball only.

### Apparatus

#### Ball test

**Fig 2:**
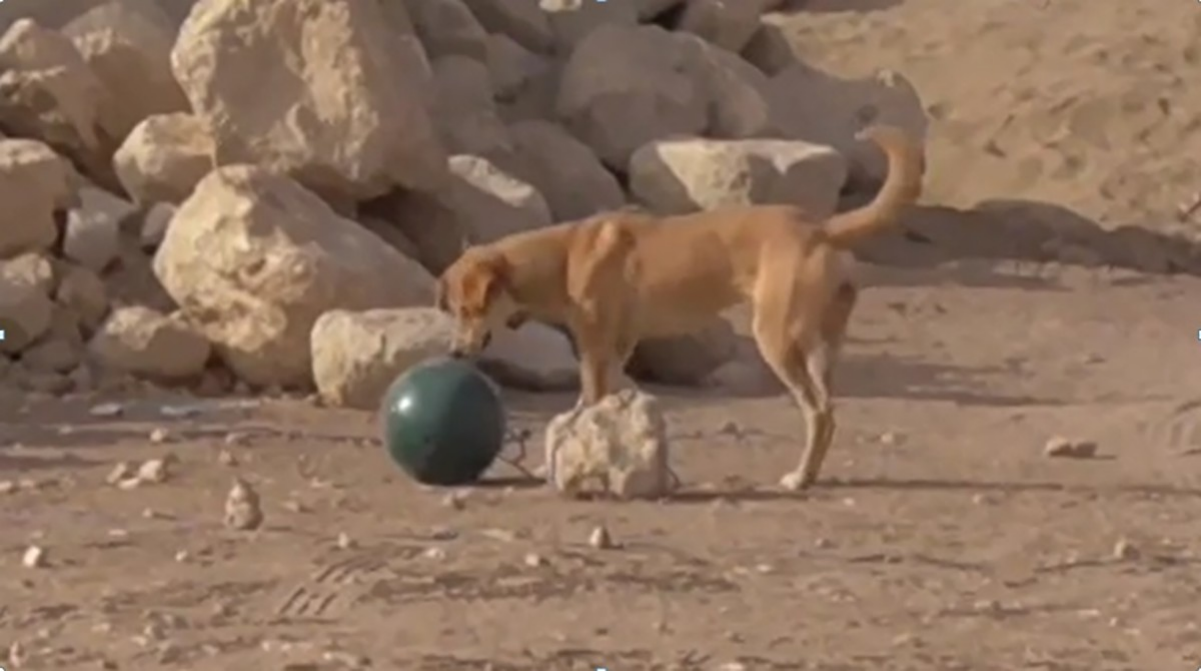
Ball.

A Lion Feeder Ball (a perforated, hard plastic sphere 24 cm in diameter, weighing 1.5 kg, commercially available “Lion Feeder Ball” from www.ottoenvironmental.com; Fig 2) baited with food impossible to reach was fixed on the ground with a 30 cm chain or a rope.

#### Bottle test

**Fig 3:**
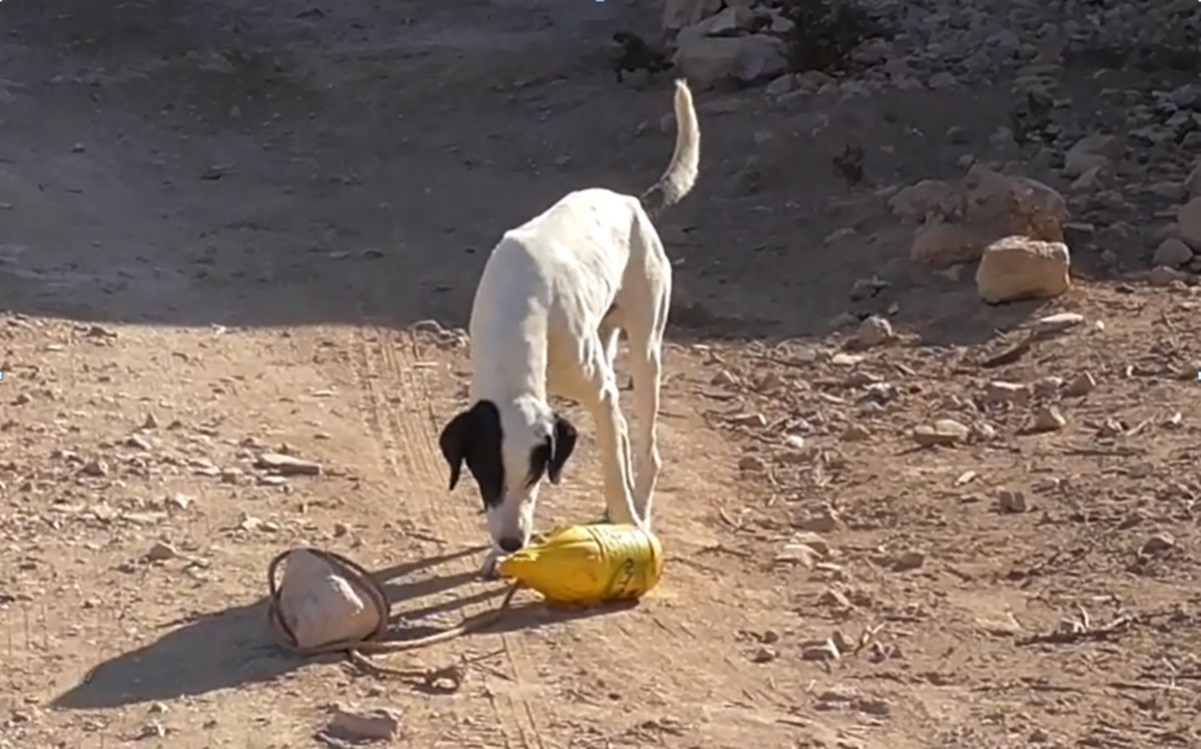
Bottle.

An opaque and rigid plastic bottle (a perforated, hard plastic bottle 30 cm length, weighing 0.25 kg; Fig 3) baited with food impossible to reach was fixed on the ground with a 30 cm rope.

The apparatuses were baited with sausage and meat for WSCd and sausage and cheese for Pd and FRd. The order of the two tests that were performed with the same subjects was randomized.

### Test procedure

We defined persistence as task-directed motivation. We were interested in measuring and comparing subjects’ persistence in their specific familiar home environment. To achieve this, we tested each dog population in their home territory, without other dogs, and in the absence of any salient stimuli (i.e. unusual for that population) (for additional statistical comparisons of the effect of testing location in Pd see S1 File).

#### Free-ranging dogs (FRd)

Free-ranging dogs were tested in the streets and on the beaches of Taghazout. The tests were done mainly in the early morning, reducing the likelihood of salient stimuli and possible interference with the test. Once a subject was located alone, the experimenter placed the apparatus on the ground, taking care to not be seen by the subject. The apparatus was anchored using a 30-cm long rope to a stone. The experimenter then hid in the car and waited for the dog to independently find the object. If after five minutes, the dog did not find the apparatus (12 dogs did not find the ball and 12 did not find the bottle), a second experimenter went to the dog, greeted it for a few seconds and then walked towards the apparatus with the subject following her. The experimenter did not indicate the apparatus to the dog but simply walked past it and then got into the car. The test started when the dog approached the apparatus. The tests were filmed from the car (S1 Video and S2 Video).

#### Pet dogs (Pd)

Pet dogs were tested in their home gardens in Austria. Once the experimenter arrived at the house, the animals were taken inside. Then the experimenter placed two cameras and the apparatus on the ground in the garden without being seen by the subject. The apparatus was anchored using a 30-cm long rope to a camping peg driven into the ground. Once the object was in place, the experimenter left, and the dogs were allowed into the garden. The owner and the experimenter remained in the house and were out of sight of the test subject, but able to observe the test from the house and therefore end the test at the appropriate moment.

#### WSC dogs (WSCd)

WSC dogs were tested in their home enclosures at the Wolf Science Centre, Austria. The subjects and their pack were first shifted out from their home enclosure. Before a test session began, we anchored the ball using a 30-cm long metal chain to a camping peg driven into the ground in the subjects’ home enclosure. This was done out of sight of the test subject. The peg was positioned such that we could record any interactions the subject had with the object from two different angles without any visual obstructions. We mounted two video cameras (one remote-controlled) on tripods outside the enclosures. Once the apparatus was in place, the focal subject was shifted back into the enclosure. No humans or other animals were present in the area or visible to the test subject. WSC animals are used to being shifted daily from their home enclosure and to be momentarily separated from other pack members. We tested the animals in their home enclosure where they are usually not tested to reduce the possibility that expecting human presence would interfere with their behaviour.

In all tests the subject was left to find the object on its own to avoid a potentially different effect of human behaviour/presence on the subjects’ behaviour in the three groups. In fact, observing the placing of the object on the ground might have increased subjects’ motivation to interact with the apparatus for Pd and WSCd, which are used to being tested and/or playing with objects presented by humans. On the contrary, free-ranging dogs might have been inhibited by the humans’ contact with the object, in their approach and interaction with it.

For Pd and WSCd a circle of two-body-lengths radius around the apparatus was drawn on the ground, while for FRd it was drawn on the videos (it was not possible to draw on the ground since this process risked the dog leaving the area or seeing the experimenter).

The subject was given 5 minutes to approach the object (defined as approaching the object within 10 cm). In case the subject did not approach within five minutes, the test session was terminated. The subjects were free to interact with the object for as long as they wanted and the test was considered finished when the animal left the two-body-lengths radius from the apparatus.

### Analyses

All the videos were coded using the software Solomon coder (100926 developed by András Péter, Dept. of Ethology, Budapest, www.solomoncoder.com). See Table 1 for definitions of the coded behaviours.

**Table 1:** Ethogram of the behaviours analysed.

**Contact latency**	Time the subject takes to reach the object (less than 10 cm distance) from a 2-body length circle.
**Interaction with the object**	
duration of sniffing	Time (seconds) the animal spends sniffing the object from less than 10 cm distance (without touching it).
duration of manipulation (persistence)	Time (seconds) the animal spends touching the object with either snout or paw.

The sum of the durations of sniffing and manipulating is defined as interaction with the object.

Inter-observer reliability was carried out with a second observer coding 20% of video data (Intra-class correlation coefficient: duration of manipulation ICC = 0.99, *F* = 4409, *p* < 0.0001; duration of sniffing ICC = 0.99, *F* = 232, *p* < 0.0001).

To investigate possible effects of neophobia, we analysed if the contact latency varied when subjects were tested with the ball (unfamiliar object) or with the bottle (familiar object). For Pd and FRd we ran the analyses on the subjects that performed both the ball and the bottle test (22 Pd, 10 FRd), comparing contact latency for the two objects for each group. Two GAMLSS models were used to evaluate the effects of object type on the contact latency in Pd and FRd. The subject ID was included as random factor. The model on contact latency in Pd was fitted with Box-Cox t distribution and validated with Inverse Gamma, Generalized Gamma and Generalized Inversed Gaussian distributions. The model on contact latency in FRd was fitted with a Generalized Beta type II distribution and validated with Gamma, ex-Gaussian and Weibull distributions. We additionally compared Pd with WSCd that were tested with the ball, since the two groups could be compared for this analysis since both were tested with the same procedure. We included in this analysis only Pd that did the ball test as the first test (13 Pd) to allow for a proper comparison with the WSCd that performed only the ball test and not the bottle test. A GAMLSS model was used to evaluate the effect of group (Pd vs WSCd) on the contact latency for the ball test. The model was fitted with an Inverse Gamma distribution and validated with Box-Cox Power Exponential, Inverse Gamma and Box-Cox Cole and Green distributions.

We then investigated differences in interaction time and persistence between the groups. A GAMLSS model was used to evaluate the effect of group (FRd, Pd, WSCd) on the interaction time with the ball (sum of duration of sniffing and manipulation). To compare Pd and FRd with WSCd that were tested only with the ball, we included in this model Pd and FRd that did the ball test as the first test or that were only tested with the ball (13 Pd and 13 FRd). The model was fitted with a Box-Cox t distribution and validated with Inversed Gamma, Generalized Gamma and Generalized Beta type 2 distributions.

We investigated the difference in the occurrence of manipulating the ball between groups with a general linear model (GLM) with a quasibinomial distribution. The occurrence of manipulating the ball (manipulating vs non-manipulating) was included as the response variable and group as the explanatory factor. In this analysis, we included all subjects tested with the ball that approached and sniffed the object (23 Pd, 22 FRd, 16 WSCd). Since as a group FRd were not manipulative with the ball (only 3 dogs manipulated the object), we excluded them from further analyses on the persistence in manipulating the ball and compared only Pd and WSCd. A GAMLSS model was used to evaluate the effects of the explanatory factor group (Pd, WSCd) on persistence in manipulating the ball. Since WSCd were only tested with the ball, to compare the two groups, we included in this model only Pd that did the ball test as the first test (13 Pd). We excluded the subjects that did not manipulate the ball (2 WSCd). The model was fitted with an Inverse Gamma distribution and validated with Generalized Gamma, Generalized Inverse Gaussian and Box-Cox t distributions.

We finally investigated differences in interaction time and persistence between Pd and FRd between ball and bottle test. These analyses were run on all subjects, including those that performed only one test (32 FRd: 20 ball tests and 22 bottle tests; 25 Pd: 23 ball and 23 bottle tests). In fact, a preliminary analysis showed no effect of the order of presentation or number of objects presented on the animals’ interaction time (S2 File). We excluded 1 Pd and 1 FRd that in the bottle test managed to open the object and eat the content. A GAMLSS model was run to evaluate the effects of the explanatory factors group (Pd-FRd) and object type (ball-bottle) on the response variable interaction time. We included the subject as a random factor. The model was fitted with a Box-Cox t distribution and validated with Gamma, Generalized Beta type II, Generalized Gamma distributions. We investigated the differences in the occurrence of manipulating the bottle between Pd and FRd with a general linear model (GLM) with a binomial distribution. The occurrence of manipulating the bottle (manipulating vs non-manipulating) was include as the response variable and group as the explanatory factor. In this analysis we included all subjects tested with the ball that approached and sniffed the object (23 Pd, 22 FRd). In contrast to the ball, the majority of FRd manipulated the bottle. Thus, we investigated differences in persistence between Pd and FRd for the bottle test only. A GAMLSS model was run to evaluate the effects of group (Pd-FRd) on the response variable persistence. We excluded the subjects that did not manipulate the bottle (3 Pd, 7 FRd). The model was fitted with an Exponential distribution and validated with Weibull, Pareto Type 2 and Generalized Inverse Gaussian distributions.

All models were run in the program R (version 3.4.4). We used generalized linear models (GLM) and generalized linear mixed models (GLMM). The models were fitted using the functions glm (R package stats) and glmer (R package lme4) [62, 63]. P values for the individual effects were based on likelihood ratio tests comparing the full model with the respective reduced models [64]. We additionally used the package GAMLSS (“gamlss” version 5.0–6) [65]). We used the “gamlss.Dist” package version 5.0–4 to fit distributions to our data. We validated our models’ results by fitting identical models with other probable distributions. We evaluated all models fit both by their generalised Akaike information criteria [66] and by the distribution of the model residual quantile-quantile plots. This approach enabled us to analyse the data without major transformations, which could have affected the interpretation of results [67, 68].

## Results

There was no significant effect of object type (GAMLSS: *t* = 0.09, *p* = 0.92) on the contact latency in Pd (ball: mean 1.76 s, range 0.6–8.4 s; bottle: 2.06 s, range 0.6–10.2 s). There was also no significant effect of object type (GAMLSS: *t* = -1.44, *p* = 0.17) on the contact latency in FRd (ball: mean 2.64 s, range 0.2–7.6 s; bottle: mean 2.08 s, range 1–5.2 s). WSCd were found to be faster than Pd in approaching the ball (GAMLSS: *t* = -4.3, *p* < 0.001) (WSCd mean 0.98 s, range 0.6–1.2 s; Pd mean 2.38 s, range 0.6–8.4 s).

**Fig 4:**
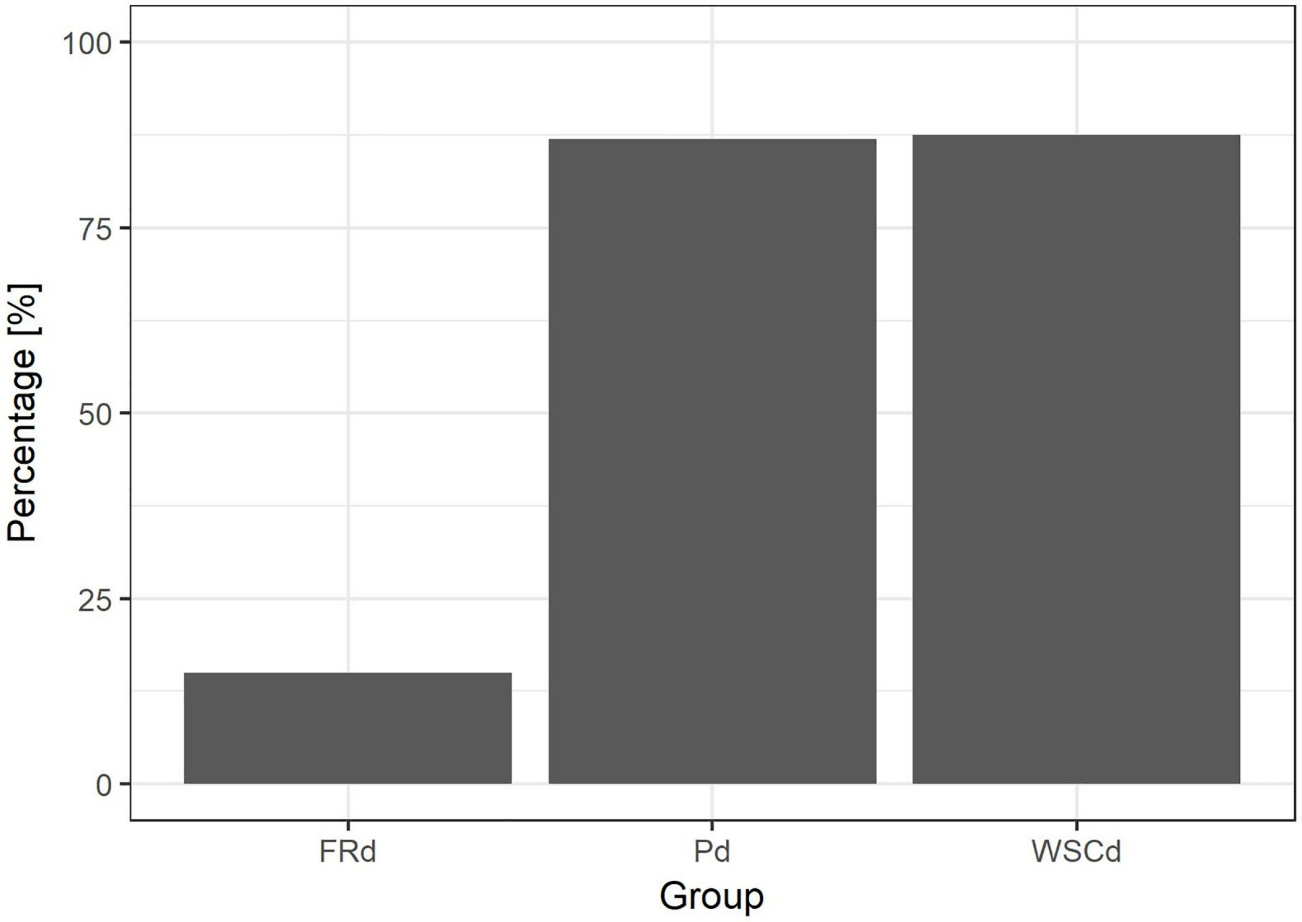
Manipulation of the ball. Proportion of individuals that manipulated the ball in the three groups (Pd: pet dogs, FRd: free-ranging dogs, WSCd: WSC dogs).

**Fig 5:**
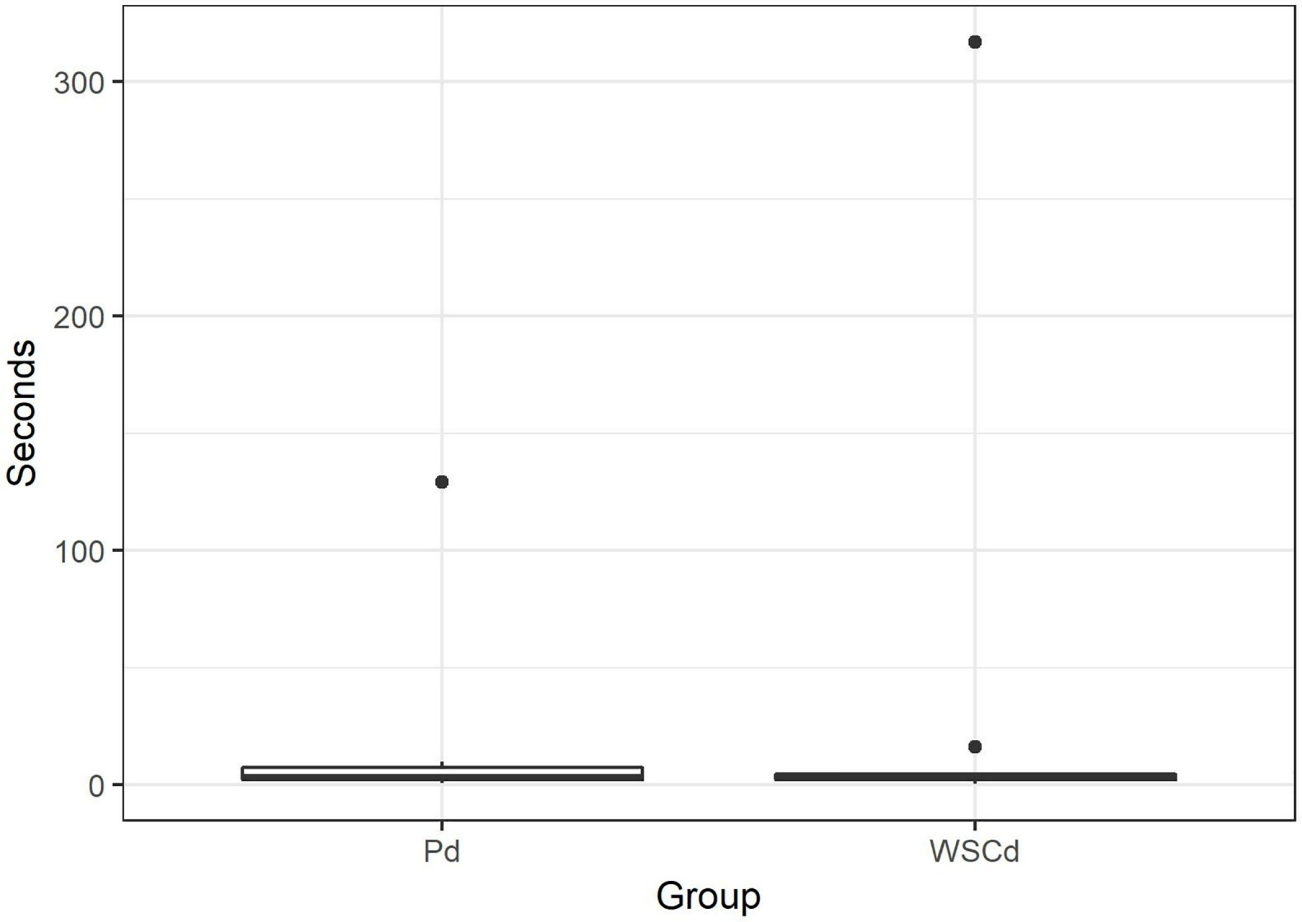
Duration of manipulation of the ball for pet dogs (Pd) and WSC dogs (WSCd).

The three groups did not differ in the interaction time with the ball (i.e. interacting: sum of sniffing and manipulating) (GAMLSS: FRd-Pd *t* = 1.2, *p* = 0.23; FRd-WSCd *t* = 0.34, *p* = 0.73; Pd-WSCd *t* = -0.91, *p* = 0.36) (FRd mean 15.64 s, range 5.6–39 s; Pd mean 30.04 s, range 3.4–147.6 s; WSCd mean 32.4 s, range 6–337 s). Despite all FRd that approached the ball also sniffed it, only 3 manipulated it. Thus, analysing whether the subjects manipulated the ball or not, we found significant difference between FRd and Pd (GLM: *z* = 4.02, *p* < 0.001) and FRd and WSCd (GLM: *z* = 3.6, *p* < 0.001), while no differences were observed between Pd and WSCd (GLM: *z* = 0.05, *p* = 0.9), Fig 4. We did not find differences in persistence in manipulating the ball between Pd and WSCd (GAMLSS: *t* = -0.56, *p* = 0.58) (Pd mean 13.9 s, range 0.8–129.4 s; WSCd mean 26.18 s, range 0.8–316.8 s), Fig 5.

**Fig 6:**
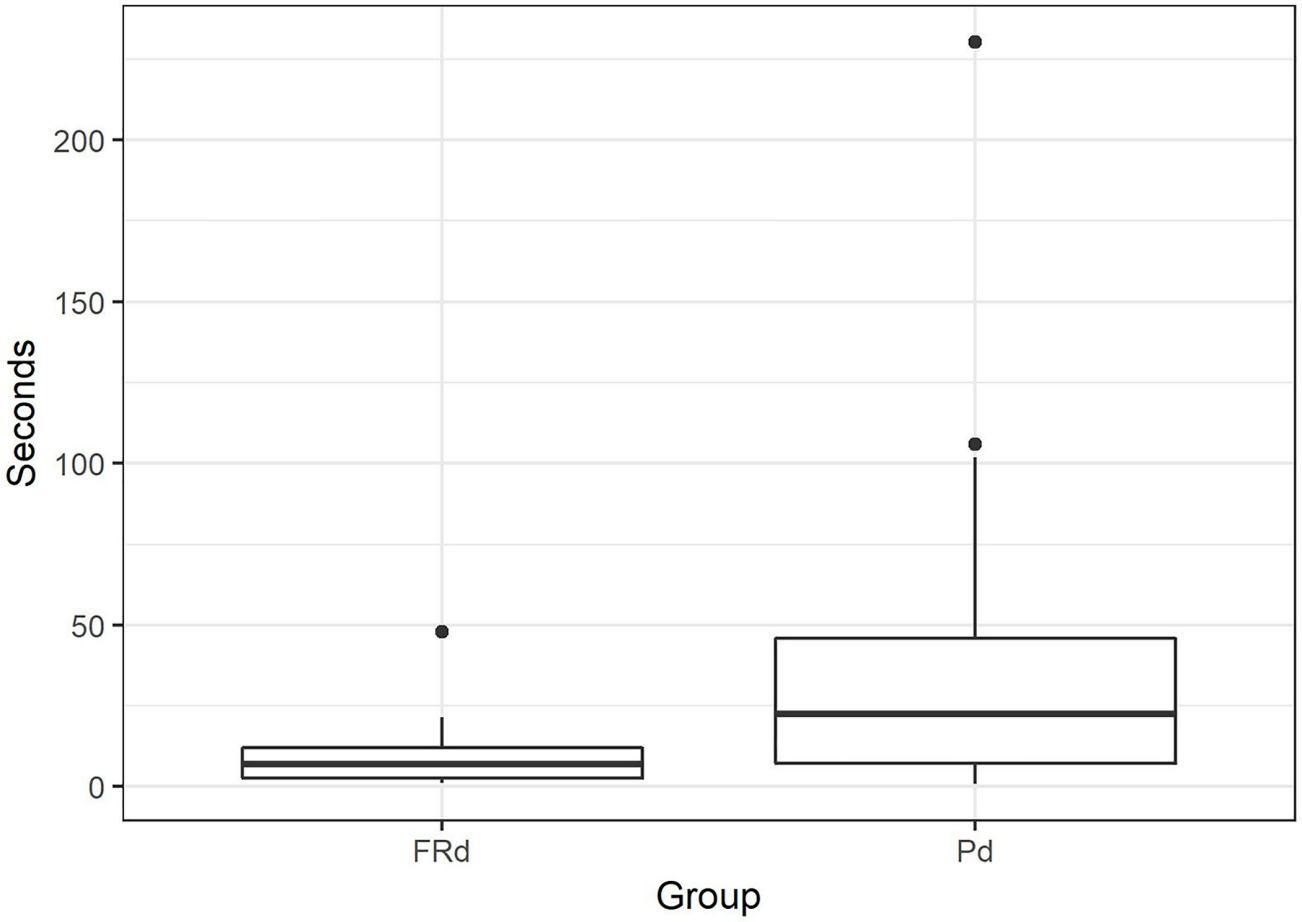
Duration of manipulation of the bottle for free-ranging dogs (FRd) and pet dogs (Pd).

Pd and FRd were additionally tested with a bottle representing a familiar object for these two populations. There was no significant effect of group (Pd-FRd) (GAMLSS: *t* = 0.86, *p* = 0.4) or object type (ball-bottle) (GAMLSS: *t* = 0.57, *p* = 0.57) on interaction time (ball: Pd mean 33.97 s, range 2–147.6 s; FRd mean 15.14 sec, range 3.8–44.8 s; bottle: Pd mean 33.78 s, range 2.4–112.8 s; FRd mean 15.61 s, range 6–64.6 s). The interaction between group and object type was not significant (*t* = 0.5, *p* = 0.62). Differently from the ball test, in the bottle test FRd manipulated the object and did not differ from Pd (GLM: *z* = 1.47, *p* = 0.14). However, FRd were less persistent than Pd in manipulating the bottle (GAMLSS: *z* = 3.17, *p* = 0.003), (Pd mean 42.5 s, range 0.8–230.2 s; FRd mean 10.8 s, range 1–47.8 s), Fig 6.

## Discussion

We investigated differences in persistence in an impossible task in three groups of dogs with different life experiences. We found that pet dogs and captive dogs were more manipulative and persistent than free-ranging dogs. This is in contrast with the common thinking that pet dogs are bad problem solvers due to owners training them to inhibit object manipulations and/or the lack of needing to independently solve tasks.

We suggest that the lower manipulation and persistence of free-ranging dogs compared to pet dogs and WSC dogs cannot be attributed to greater neophobia towards the objects. In fact, for free-ranging dogs we could not find differences in latency to approach a novel object (ball) and a familiar object (bottle). Unfortunately, we could not directly compare the latency to approach the ball between free-ranging dogs and the other groups, since they were tested with different procedures (random starting point for free-ranging dogs compared to a fixed starting point for the other groups). Free-ranging dogs usually roam around slowly looking for food, while WSC dogs or pet dogs usually run into their enclosures/gardens engaging in exploration activities (pet dogs) or looking for a reward (WSC dogs). However, we did compare pet dogs and WSC dogs and found that the latter were faster in approaching the object despite being equally persistent. This was probably because WSC animals are used to being shifted out from their home enclosures and then receive treats or enrichment once shifted back in, which might increase their motivation and speed in moving back into the enclosure.

Despite free-ranging dogs being particularly less manipulative than pet dogs, the two groups did not differ in their overall interest toward the objects, interacting similarly with the bottle and the ball. This additionally supports the absence of neophobia in free-ranging dogs towards the objects, compared to pet dogs. However, both groups were more persistent when manipulating the bottle than the ball. These results are likely due to different features of the objects that could have affected subjects’ persistence, since the bottle appeared to be easier to manipulate than the ball [69].

We suggest two possible explanations as to why free-ranging dogs were less persistent than pet dogs and WSC dogs: 1) free-ranging dogs may be less willing to persist in a difficult task than pet dogs and WSC dogs to conserve energy [70]; 2) experiential factors differing between groups may have influenced subjects’ persistence in the task, e.g., different training experiences with objects–both the WSC dogs and pet dogs are encouraged by the trainers/owners to interact with objects in certain situations (i.e. experiments, dog toys).

In regard to the first explanation, we observed that in the study area, where we tested free-ranging dogs, the food distribution is variable. Thus, in some locations the food sources are predictable, either concentrated or scattered, while in other locations the food sources are unpredictable, which is likely connected to the variability of human activity. This food distribution results in solitary dogs roaming around looking for food, moving from one spot to another to eat scattered garbage. In accordance with the optimal foraging strategy, since the subjects might perceive our task as extremely difficult if not impossible, the best foraging strategy would result in ignoring the task and looking for other food sources [71]. This strategy would be additionally supported by the fact that the other food sources are easy to locate and consume and have high energetic value. While free-ranging dogs are used to finding scattered food, pet dogs and WSC dogs are used to predicable food located in a single spot. Thus, free-ranging dogs may not waste energy in trying to obtain food in one location but decide to move to the next spot because other food resources are usually available. Moreover, they may have learned that if they cannot get access to food within human artefacts easily, it is more energy efficient to move on and look for other sources of food, which might otherwise be found by competing scavengers. However, in contrast with this interpretation is the observation that many subjects (21.7%) laid down close to the apparatus or continued standing in the area without looking for other food sources. Moreover, the presence of other dogs increased the subjects' interaction time with the ball. In fact, an additional 13 dogs where tested for this study, but excluded because another dog appeared either at the start or during the test. Comparing these dogs to the study sample of 23 dogs tested alone we found that dogs tested with another dog present interacted with the ball longer. Furthermore, when testing the time spent interacting with the ball before compared to after the second dogs’ arrival, we found that the arrival of a dog tended to increase subjects’ interaction time with the object (S3 File).

Another possible factor that might have determined the differences in persistence between dog groups is their different experiences with humans. Interestingly, the comparison between the three dog groups allowed us to refute the hypothesis that dogs' low persistence may be due to a conditioned inhibition of problem-solving behaviour [53]. In fact, neither free-ranging dogs nor WSC dogs experience the inhibitory influence of a human companion controlling their potentially destructive tendencies in a home environment. Yet WSC dogs were just as persistent as pet dogs and free-ranging dogs were less persistent than the other groups. Rather, our results are in line with findings from other studies that compared subjects in captivity and in the wild and found contact with humans to increase problem-solving success [20–22]. Although these studies did not measure persistence *per se*, problem-solving success and persistence have often been observed to be positively correlated [12–14, 72–75]. Though the increased success of captive animals is commonly attributed to their greater chances to interact with human objects during their lifetime, this was not so in our case. In fact, in our study area, free-ranging dogs live around human settlements and are always in contact with garbage disposed in the streets and open-air dumps. This often includes discarded containers of various kinds and provides dogs with many opportunities to interact with human artefacts. Additionally, the presence of food around or inside the variety of objects that can be found, points to the possibility that free-ranging dogs may have associated human artefacts with food just as much as the other two dog groups. Thus, we suggest that a possible factor influencing the difference in persistence observed between free-ranging dogs and pet dogs-WSC dogs could be the experience of humans mediating the animals’ interaction with the objects around them. In fact, although it has been observed that the mere presence of a human does not increase the dog’s persistence [53], the human’s encouragement does [52, 53]. Thus, the life experience of object-interaction mediated by human encouragement (through play and/or positive social response) may increase the subjects’ overall motivation to interact with objects. This would also explain why pet dogs and WSC dogs showed similar persistence levels: despite living in such different environments, both groups experience human-guided interactions with objects. In support with this interpretation are the findings of studies comparing trained and untrained pet dogs in problem-solving tasks (belong to the same pet dog population, having similar food motivation and health conditions) that have consistently found trained dogs, regardless of the training discipline (agility, search and rescue, dog dancing etc.), to be more persistent and have higher problem-solving success than untrained subjects ([48–51] but see [52]).

To sum up, we observed that free-ranging dogs were less persistent than pet dogs and pack dogs living in enclosures. This is in contrast with the common thinking that pet dogs are inhibited to interact with objects and/or do not need to do it because they are used receiving help from a human partner. Although further studies are necessary to deepen our understanding of the reason underlying these differences, we suggest that a possible explanation for this finding is the different human-mediated object interaction between groups. Humans may provide subjects with a socially guided experience in manipulating and interacting with objects, which could increase their motivation to engage in such tasks (even in their absence). Thus, pet dogs as well as pack-living captive dogs, both with ample experience of human-mediated object interaction, spent significantly longer manipulating the object than free-ranging dogs. This is in line with observations in other species when comparing captive and wild subjects. Finally, these results support the findings of a previous study focusing on a wolf-dog comparison suggesting that the lower persistence of dogs when compared to wolves, is a consequence of the different foraging ecologies of the two species (hunting vs scavenging) rather than the effect of humans inhibiting dogs' interactiveness with objects [57].

## Supporting information

S1 File Additional analyses in pet dogs on the possible effect of testing location on interaction time and persistence. (DOCX)

S2 File Additional statistics comparing Pd and FRd that carried out both ball and bottle test. (DOCX)

S3 File Additional statistics comparing free-ranging dogs tested alone and in the presence of other dogs, in the ball test. (DOCX)

S1 Video Free-ranging dog tested with the ball. (MP4)

S2 Video Free-ranging dog tested with the ball. (MP4)

S1 Data Data used for the analyses. (XLSX)
