# Supplementary material for: The role of life experience in affecting persistence: A comparative study between free-ranging dogs, pet dogs and captive pack dogs
Source: PLoS One. 2019 Apr 17;14(4):e0214806. doi: 10.1371/journal.pone.0214806 (PMC6469757; doi:10.1371/journal.pone.0214806)
Supplement: S1 File — (DOCX) [file pone.0214806.s001.docx]

**S1 FILE.**

**Additional analyses in pet dogs on the possible effect of testing location on interaction time and persistence.**

We initially ran a pilot study on 10 Pd in an outdoor area with both the ball and the bottle. The test procedure was identical to the one used for Pd in the current study, except for the fact that the owner was present in the dog area, sitting on a chair hidden behind a tree. No other dogs were present in the dog area or in sight of the subject. All the subjects were left in the dog area for 5 minutes before the test started. We did not include these subjects in the main study because the presence of the owner might have strongly affected the persistence of the subjects [1], thus possibly affecting the comparison between Pd tested with the owner and FRd and WSCd tested alone.

However, we investigated a possible effect of the test location (garden vs dog area) on interaction time and persistence in Pd. We run two GAMLSS models to evaluate the effects of the explanatory factors test location and object type (ball and bottle) on the interaction time or on persistence. The subject was included as a random factor. We included in these analyses only Pd that were tested in their home gardens with both the ball and the bottle test (22 Pd) and 10 different Pd tested in an outdoor area with both objects. We removed from the analyses one Pd in the bottle test, tested in the garden, that opened the bottle eating the content. We additionally excluded the subjects that did not manipulate the ball (4 subjects tested with the ball in the dog area, 3 subjects tested with the ball and 3 subjects tested with the bottle in the gardens). The model on interaction time was fitted with an Inversed Gamma distribution and validated with Log Normal, Generalized Inverse Gaussian and Generalized Gamma distributions. The model on persistence was fitted with a Generalized Inverse Gaussian distribution and validated with Log Normal, Pareto type 2 and Generalized Gamma distributions.

There was no difference in the interaction time with the objects between Pd tested in the gardens and Pd tested in the outdoor area (GAMLSS: *t* = 0.08, *p* = 0.93) nor difference in the interaction time with the two objects (GAMLSS: *t* = 1.78, *p* = 0.08). There was no difference in persistence with the objects between Pd tested in the gardens and Pd tested in the outdoor area (GAMLSS: *t* = 0.89, *p* = 0.37), but independently from the area where the dogs were tested, subjects were more persistent in manipulating the bottle than the ball (GAMLSS: *t* = 2.36, *p* = 0.02).

These results suggest that the testing location did not have an influence on subjects’ interaction time and persistence with the objects. However, we could not exclude that the presence of the owner might have differently influenced subjects’ behaviours. Anyhow, in this case we would have expected that the presence of the owner would have reduced subjects’ persistence as suggested in previous studies [1, 2].

1. Udell MA. When dogs look back: inhibition of independent problem-solving behaviour in domestic dogs (*Canis lupus familiaris*) compared with wolves (Canis lupus). Biology Letters. 2015;11(9): 20150489.

2. Topál J, Miklósi Á, Csányi V. Dog-human relationship affects problem-solving behavior in the dog. Anthrozoös. 1997;10(4): 214-224.
