# Supplementary material for: The role of life experience in affecting persistence: A comparative study between free-ranging dogs, pet dogs and captive pack dogs
Source: PLoS One. 2019 Apr 17;14(4):e0214806. doi: 10.1371/journal.pone.0214806 (PMC6469757; doi:10.1371/journal.pone.0214806)
Supplement: S2 File — (DOCX) [file pone.0214806.s002.docx]

**S2 FILE.**

**Additional statistics comparing Pd and FRd that carried out both ball and bottle test.**

We investigated if the test order (test done as first or as second) influenced the interaction time with the objects in both Pd and FRd. For this statistic we considered only the subjects that did both tests (10 FRd, 22 Pd). We run a GAMLSS model to evaluate the effects of the explanatory factor test order (test done as first or as second) on the response variable interaction time. We included the animal ID as a random factor. The model was fitted with a Box-Cox-t distribution and validated with Gamma, Generalized Beta type II, Generalized Gamma distributions. We found that the order in which the subjects performed the tests did not influence the time spent interacting with the objects (GAMLSS: *t* = -1.02, *p* = 0.312) (the interaction group*tests order was not significant (GAMLSS: *t* = 0.43, *p* = 0.668). We additionally investigated whether the number of tests performed by the subjects influenced the time spent in interacting with the objects in FRd. For this statistic we considered all 32 FRd tested. We run a GAMLSS model with test number (1 or 2 tests) as explanatory factor and interaction time as response variable. The model was fitted with a Box-Cox-t distribution and validated with Gamma, Generalized Beta type II, Generalized Gamma distributions. We found that the number of tests performed by the subjects (1 or 2 tests) did not influence the time spent interacting with the object (GAMLSS: *t* = -1.36, *p* = 0.18).
