# Supplementary material for: The role of life experience in affecting persistence: A comparative study between free-ranging dogs, pet dogs and captive pack dogs
Source: PLoS One. 2019 Apr 17;14(4):e0214806. doi: 10.1371/journal.pone.0214806 (PMC6469757; doi:10.1371/journal.pone.0214806)
Supplement: S3 File — (DOCX) [file pone.0214806.s003.docx]

**S3 FILE.**

**Additional statistics comparing free-ranging dogs tested alone and in the presence of other dogs, in the ball test.**

While running the test with free-ranging dogs, we tested 13 additional dogs with the ball, which were excluded from the main study because during the test another dog (initially not present) approached. Although we did not specifically design the study to test the differences in the interaction time with the object between dogs tested alone and dogs tested in the presence of other dogs, we analysed these data, since it may help clarify the motivation for the reduced persistence in free-ranging dogs. Nevertheless, these results should be considered carefully, and further studies should be run to properly test differences in interaction with objects in the presence or absence of other dogs.

We investigated if the presence of other dogs influenced the overall interaction time with the ball. For this statistic we considered 23 FRd tested alone and 13 FRd tested with the presence of other dogs. We run a GAMLSS model to evaluate the effects of the explanatory factor presence of other dogs on the response variable interaction time. The model was fitted with Zaga distribution. We found that subject tested with other dogs interacted significantly longer with the ball than subjects tested alone (GAMLSS: *t* = 2.24, *p* = 0.03). We additionally investigated whether the arrival of the other dogs influenced the time spent in interacting with the ball. For this statistic we considered the 13 FRd tested in the presence of other dogs. We run a GAMLSS model with before/after the arrival of the other dog as explanatory factor and interaction time as response variable. Subject was included as random factor. The model was fitted with a Zaga distribution. We found that after the arrival of another dog, subjects tended to increase their interaction time with the object (GAMLSS: *t* = -1.9, *p* = 0.06).
